# Resuscitative transesophageal echocardiography in the diagnosis of post-CABG loculated pericardial clot causing cardiac tamponade

**DOI:** 10.1186/s13089-021-00225-7

**Published:** 2021-04-15

**Authors:** Osman Adi, Azma Haryaty Ahmad, Chan Pei Fong, Asri Ranga, Nova Panebianco

**Affiliations:** 1 Resuscitation & Emergency Critical Care Unit, Department of Emergency and Trauma, Raja Permaisuri Bainun Hospital, Jalan Raja Ashman (Jalan Hospital), 30400 Ipoh, Perak Malaysia; 2 Department of Cardiology, Hospital Serdang, Serdang, Selangor Malaysia; 3 Division of Emergency Ultrasound, Department of Emergency Medicine, Hospital of the University of Pennsylvania, 3400 Spruce St, Philadelphia, PA 19104 USA

**Keywords:** Resuscitative transesophageal echocardiography, Post-CABG posterior pericardial clot, Cardiac tamponade

## Abstract

**Background:**

Pericardial effusion is a known complication of post-open cardiac surgery which can progress to life-threatening cardiac tamponade. Classical signs of tamponade such as hypotension and pulsus paradoxus are often absent. Diagnosing acute cardiac tamponade with transthoracic echocardiography (TTE) can be challenging in post-cardiac surgical patients due to distorted anatomy and limited scanning windows by the presence of surgical dressings or scar. Additionally, this patient population is more likely to have a loculated pericardial effusion, or an effusion that is isoechoic in appearance secondary to clotted blood. These findings can be challenging to visualize with traditional TTE. Missed diagnosis of cardiac tamponade due to loculated pericardial clot can result in delayed diagnosis and clinical management.

**Case presentation:**

We report a case series that illustrates the diagnostic challenge and value of resuscitative transesophageal echocardiography (TEE) in the emergency department (ED) for the diagnosis of cardiac tamponade due to posterior loculated pericardial clot in post-surgical coronary artery bypass graft (CABG) patients.

**Conclusions:**

Cardiac tamponade due to loculated posterior pericardial clot post-CABG requires prompt diagnosis and appropriate management to avoid the potential for hemodynamic instability. Transesophageal echocardiography allows a rapid diagnosis, early appropriate referral and an opportunity to institute appropriate therapeutic measures.

**Supplementary Information:**

The online version contains supplementary material available at 10.1186/s13089-021-00225-7.

## Background

Pericardial effusion is a known complication of open cardiac surgery with 1–3% of patients needing intervention [1–3]. Postoperative pericardial effusion secondary to bleeding into the pericardial sac is commonly identified prior to hospital discharge [1]. Delayed pericardial effusion can arise within a week to 6 months post-cardiac surgery [4, 5]. The progression of this collection as a cause of life-threatening cardiac tamponade can be overlooked because of atypical symptoms and absence of classical echocardiography changes [5–7].

Late-onset pericardial effusion is commonly related to post-pericardiotomy syndrome due to inflammation of the pericardium after a cardiac procedure [8]. Loculated effusions are more common in this patient population because of pericardial adhesions or scarring associated with surgery [6, 9]. Inflammation or bleeding may lead to fluid accumulation at the posterior and lateral part of the pericardium, making it difficult to visualize with transthoracic echocardiogram (TTE) [10, 11]. Transthoracic echocardiogram has been identified as a sensitive modality to evaluate the heart for complications after cardiac surgery [12].

In recent years, the usage of TEE has escalated in the emergency department (ED) and critical care, and this trend is well documented in the literature [13–22]. Focused or resuscitative TEE are terms used to describe the usage of limited TEE views to facilitate early recognition of pathologic processes and to guide clinical decision-making in critically ill patients [13–15]. In contrast to the 20-view conventional TEE in a standard cardiology exam, focused TEE utilizes five important views which are the mid-esophageal four-chamber, mid-esophageal long axis, transgastric short axis, bicaval view, and descending thoracic aorta long axis [16, 17]. Focused TEE in the ED is beneficial in the management of cardiac arrest [17, 18] undifferentiated shock [19] and trauma [20, 21]. The use of TEE by non-cardiologists in the ED and intensive care setting for critically ill patients is safe [22] and extremely useful in resource-limited setting [23]. However, focused TEE does not replace the need for comprehensive TEE when clinical questions exceed the scope of a limited exam.

In this case series, we describe the role of resuscitative TEE in managing patients with loculated cardiac tamponade post-coronary artery bypass grafting (CABG) surgery.

## Case 1

A 52-year-old male patient presented to the ED with dizziness and breathlessness. He was recently discharged after an on-pump CABG for triple vessel disease. His post-operative recovery was uneventful, and his hospital length of stay was 10 days. On representation to the ED, he was in shock with blood pressure (BP) of 84/45 mmHg, afebrile and pulse rate (PR) of 98 beats per minute with no pulsus paradoxus. He was tachypneic with a respiratory rate (RR) of 24 breaths per minute and oxygen spirometry (SpO_2_) 97% on room air. On examination, his jugular venous pressure was not elevated. His sternotomy scar was clean and not inflamed. His cardiorespiratory and abdominal examinations were unremarkable. There was no neurological deficit. Echocardiogram showed sinus rhythm without any ischemic changes. Chest X-ray showed cardiomegaly with clear lung fields. His arterial blood gas showed good gas exchange with no metabolic acidosis. The hemoglobin level was 11 g/dL and his coagulation profile was normal. The renal and liver function tests did not show any abnormalities.

**Fig. 1:**
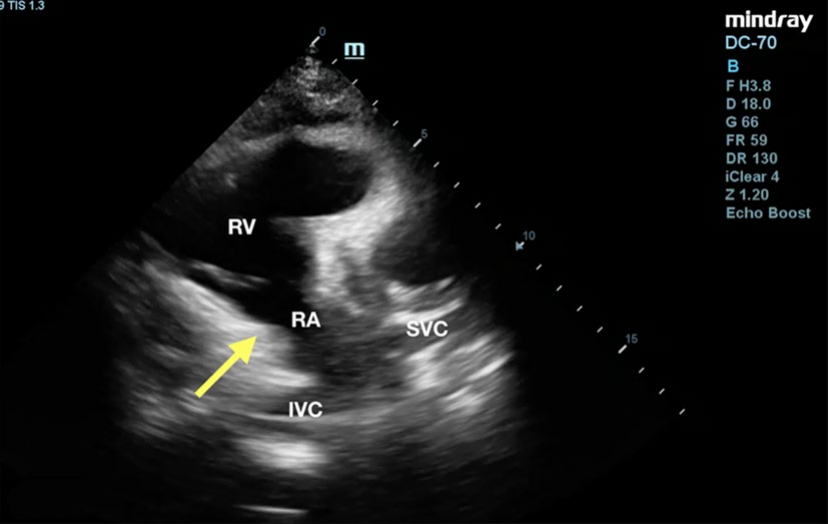
Transthoracic echocardiogram (TTE) of right ventricle inflow view showed a small and compressed right atrium (yellow arrow). Posterior pericardial clot is not well visualized in this view. *IVC* inferior vena cava, *RA* right atrium, *RV* right ventricle, *SVC* superior vena cava

**Fig. 2:**
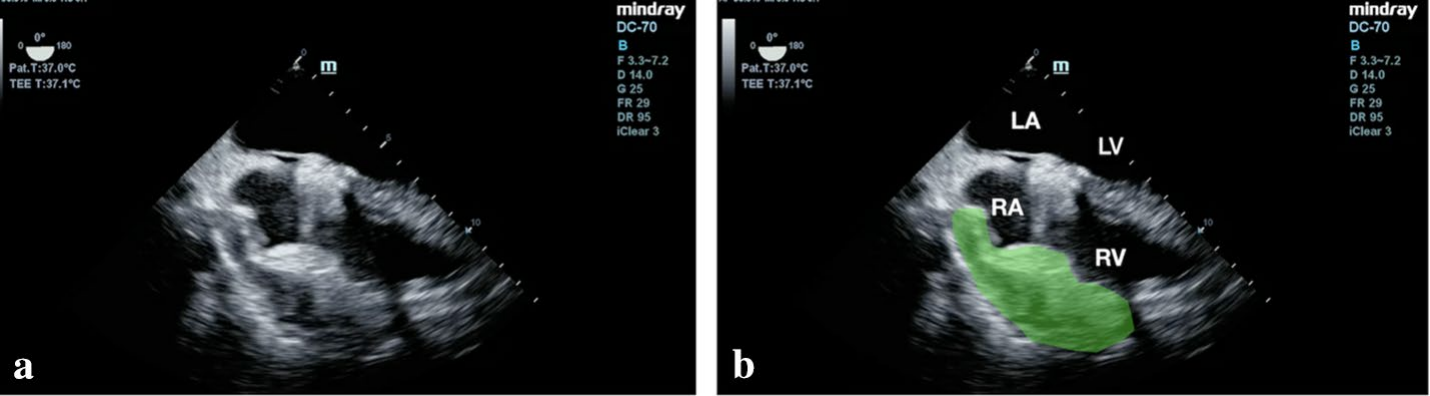
**a**, **b** Transesophageal echocardiogram (TEE) at mid-esophageal 4-chamber view with probe rotating to the right showed a compressed right atrium and a posterior pericardial clot measuring about 2 × 5 cm (green watermark). *LA* left atrium, *LV* left ventricle, *RA* right atrium, *RV* right ventricle

**Fig. 3:**
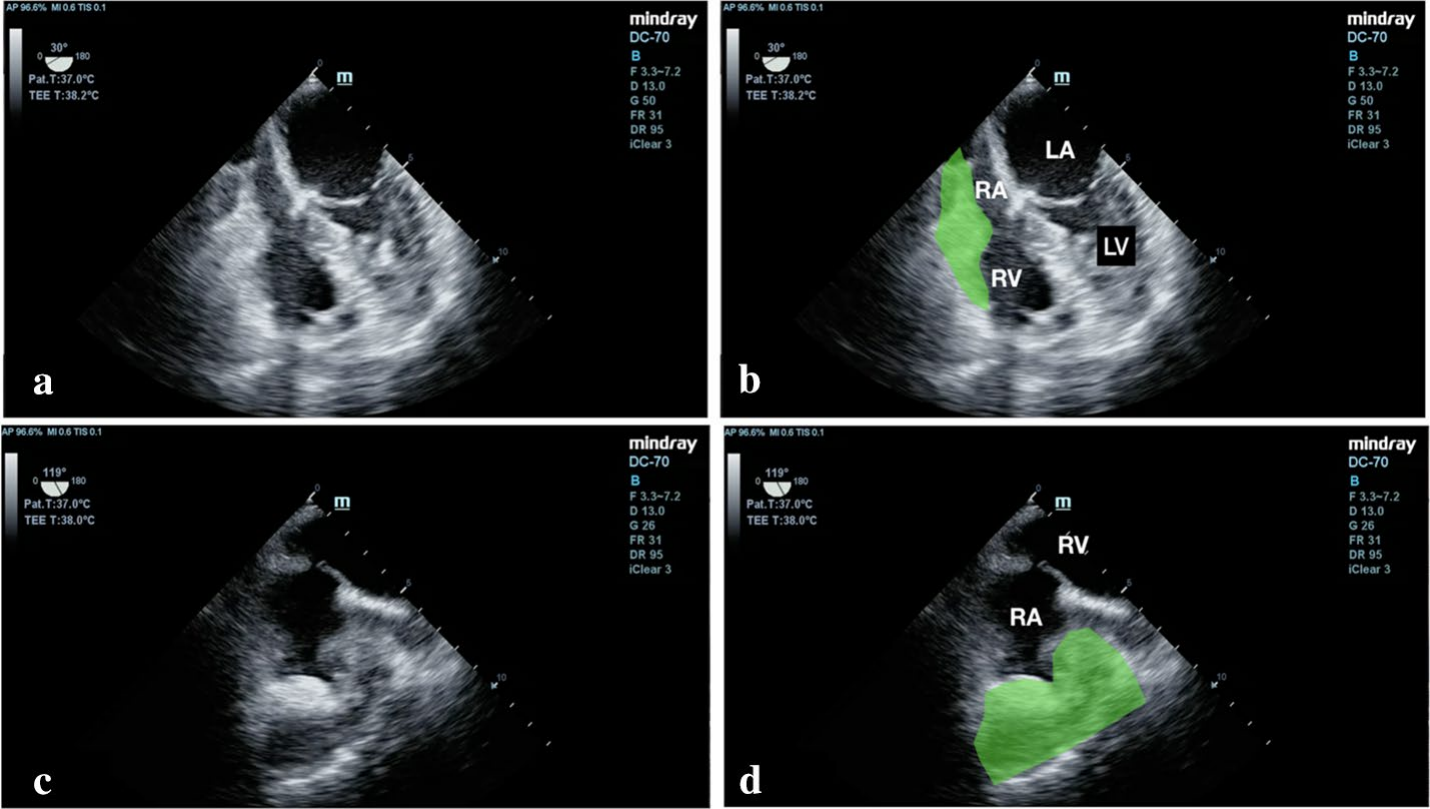
**a**, **b** Transesophageal echocardiogram (TEE) mid-esophageal 4-chamber view showed a compressed right atrium and posterior pericardial clot (green watermark). **c**, **d** TEE bicaval view showed similar findings with right atrium compression by an extrinsic posterior pericardial clot (green watermark). *LA* left atrium, *LV* left ventricle, *RA* right atrium, *RV* right ventricle

The bedside TTE of right ventricle (RV) inflow view showed compression of the right atrium (RA) by a pericardial clot (Fig. 1, Additional file 1: Video S1). Given the TTE findings, TEE was performed by the attending emergency physician and revealed a localized posterior clot measuring 3 × 5 cm compressing the right atrium (Figs. 2, 3, Additional file 2: Video S2, Additional file 3: Video S3 and Additional file 4: Video S4). He was started on intravenous infusion of noradrenaline 0.3 mcg/kg/min for blood pressure support. The patient was diagnosed with possible cardiac tamponade due to loculated posterior pericardial clot and transferred to his previous managing cardiothoracic surgery team which was 200 km away for definitive surgical intervention after cardiology consultation. He made a complete recovery and was discharged after a week.

## Case 2

A 62-year-old obese diabetic male patient presented to the ED complaining of dizziness and syncope 11 days after an on-pump CABG procedure for left main stem disease. On examination, he was drowsy and tachypneic. He was ill appearing with a BP of 75/42 mmHg, PR 88 beats per minute, without pulsus paradoxus, RR 30 breaths per minute and SpO_2_ 85% on room air. He had a short neck and his neck veins were not prominent. His midline chest wound was well healed. Lung examination showed decreased air entry at the bases. Physical examination of his other systems was unremarkable. Echocardiogram revealed a sinus rhythm without any acute ischemic changes. Cardiomegaly on chest X-ray revealed bilateral basal atelectasis. The patient was intubated for respiratory distress and hemodynamics stabilized with intravenous noradrenaline 0.2 mcg/kg/min. His arterial blood gas showed pH 7.30, PO_2_ 90 mmHg, PCO_2_ 57 mmHg, HCO_3_ 37 mmol/L, and BE 10 mmol/L under a low setting synchronized intermittent mandatory ventilation (SIMV) mode. His complete blood count was normal with hemoglobin of 14 g/dL, white blood count 10 X 10^9^/L and platelet count of 160 × 10^9^/L. Apart from a mildly raised glucose of 14 mmol/L and a baseline raised creatinine of 120 umol/L, his other blood profile values were within a normal range.

Bedside TTE images were suboptimal except for the subcostal view which showed good bi‐ventricular systolic function with trace pericardial effusion (< 1 cm). Other structures could not be evaluated with TTE due to the poor echo window.

**Fig. 4:**
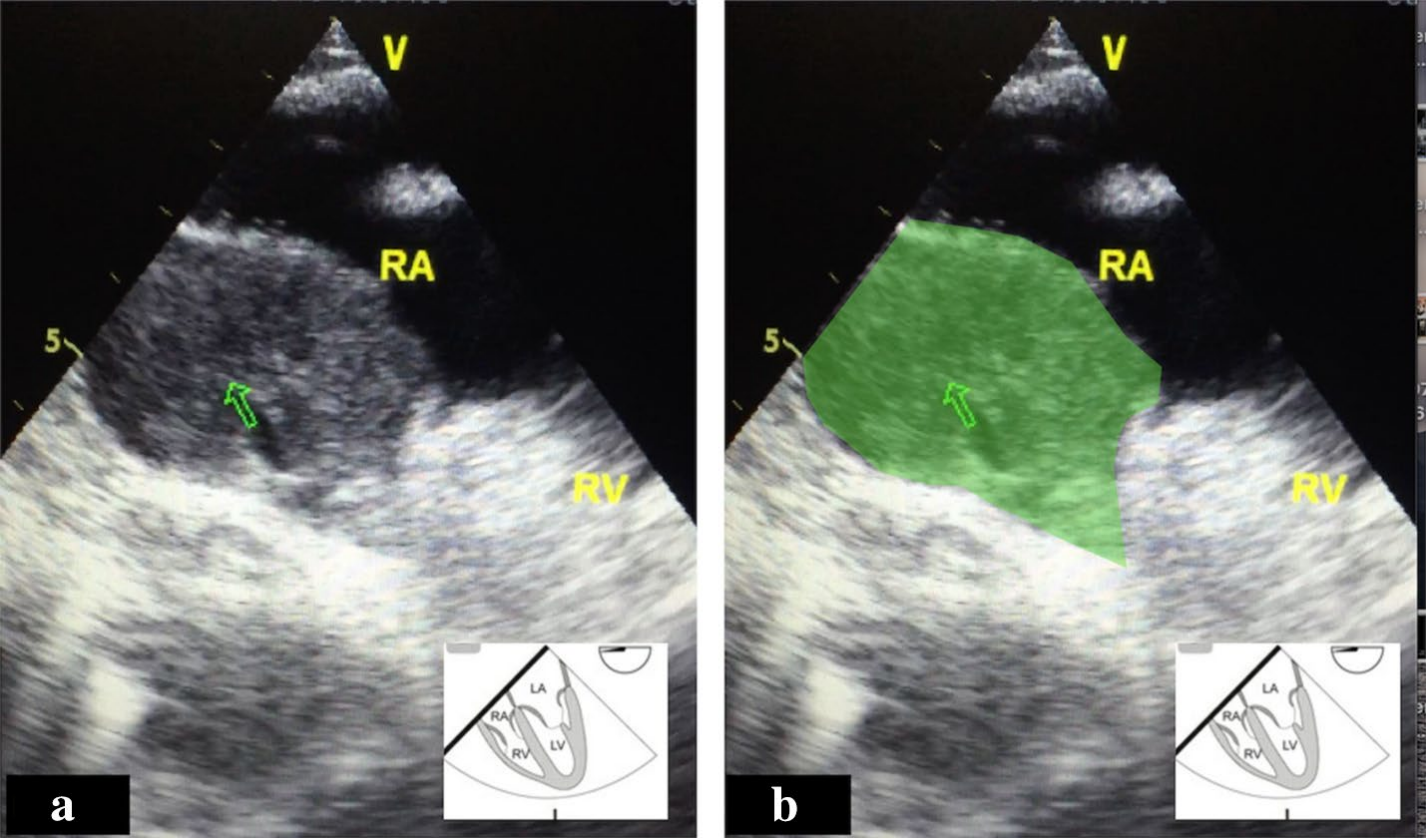
**a**, **b** Transesophageal echocardiogram (TEE) at mid-esophageal 4-chamber view with probe rotating to the right showed a compressed right atrium and a posterior pericardial clot measuring (green watermark). *RA* right atrium, *RV* right ventricle

Transesophageal echocardiography was performed in ED by attending emergency physician and revealed a large clot localized in the posterior pericardial cavity around the right atrium at the base of the heart (Fig. 4, Additional file 5: Video S5). The clot partially obliterated the right atrium during diastole causing tamponade physiology. The TEE findings were conveyed to an in-house cardiologist. The patient was readmitted to the cardiothoracic surgery for surgical drainage and he made an uneventful recovery.

## Discussion

There are several conditions that can cause hemodynamic instability in post-cardiac surgery patients, with their own unique treatment pathways. These conditions include failed graft, sepsis, bleeding, pulmonary embolism, tamponade and cardiac arrhythmias. Therefore, rapid stabilization and diagnosis are crucial for life-saving interventions to be performed. However, assessment of these undifferentiated post-cardiac surgery patients is challenging due to non-specific symptoms and signs as in the cases described earlier.

In the pathophysiology of cardiac tamponade of any cause, increased intrapericardial pressure exceeds the intracardiac pressure causing compression of the adjacent cardiac chamber [24]. Pericardial effusion after cardiac surgery may be loculated due to a pericardial adhesion that may lead to selective chamber compression [5]. Constant high left ventricular pressure in the isolated right-sided chamber compression may explain the absence of classical signs of cardiac tamponade such as pulsus paradoxus, transmitral and transtricuspid flow variability [25].

Low-pressure cardiac tamponade physiology can also occur in post-cardiac surgery patients. This is due to medication-induced reduction in filling pressures by the use of diuresis and vasodilators [24]. The suppression of the compensatory sympathetic stimulation by beta-blockers may also contribute to the atypical presentation of these patients.

Both our patients presented more than a week after CABG. Apart from the obvious signs of shock, clinical examination, conventional bedside tests such as electrocardiogram and chest X-ray did not give much clue to the cause of hypotension. Point-of-care ultrasound was used to assess the volume status, determine heart function and exclude the life-threatening diagnosis.

In the first case, the TTE image was inconclusive and showed a small right atrium with posterior hyperechoic structure suggestive of compression from the extracardiac source. With this finding, a high index of suspicion that a localized pericardial effusion was present prompted the decision to proceed with TEE. In the second case, the patient was immediately intubated and stabilized with inotropic support upon arrival at the ED.

Cardiac tamponade is a complication of post-cardiac surgery that may be difficult to assess using TTE [11, 12]. Identifying loculated pericardial effusions and clotted blood in the pericardium can be challenging [12]. Typical ultrasound features found in usual cardiac tamponade cases such as right atrial systolic collapse, right ventricular diastolic collapse, swinging heart and plethoric inferior vena cava may be absent. Transesophageal echocardiography is not limited by chest wall-related barriers and may provide life-saving diagnostic information in this patient cohort.

## Conclusion

Cardiac tamponade secondary to loculated posterior pericardial clot is a life-threatening cause of hemodynamic instability in the post-CABG patient. Transesophageal echocardiography may be of limited utility because of patient-level post-surgical variables. Transesophageal echocardiography performed in the ED can allow for a rapid diagnosis, an early referral and an opportunity to institute appropriate therapeutic measures in this challenging patient population.

## Supplementary Information

**Additional file 1: Video S1.** Transesophageal echocardiogram (TEE) of right ventricle inflow view showed a small and compressed right atrium. Posterior pericardial clot is not well visualized in this view. IVC: inferior vena cava, RA: right atrium, RV: right ventricle, SVC: superior vena cava.

**Additional file 2: Video S2.** Transesophageal echocardiogram (TEE) at mid-esophageal 4-chamber view with probe rotating to the right showed a compressed right atrium and a posterior pericardial clot measuring, about 2 × 5 cm (green watermark). LA: left atrium, LV: left ventricle, RA: right atrium, RV: right ventricle.

**Additional file 3: Video S3.** Transesophageal echocardiogram bicaval view showed right atrium compression by an extrinsic posterior pericardial clot (green watermark). LA: left atrium, LV: left ventricle, RA: right atrium, RV: right ventricle.

**Additional file 4: Video S4.** Transesophageal echocardiogram mid-esophageal 4-chamber view showed a compressed right atrium and posterior pericardial clot (green watermark)**.**

**Additional file 5: Video S5.** Transesophageal echocardiogram (TEE) at mid-esophageal 4-chamber view with probe rotating to the right showed a compressed right atrium and a posterior pericardial clot (green watermark). RA: right atrium, RV: right ventricle.

## Data Availability

The materials used during the current case series are available from the corresponding author on reasonable request.

## Abbreviations

BP: Blood pressure
CABG: Coronary artery bypass graft
CT: Computed tomography
ECG: Echocardiogram
ED: Emergency department
Fig.: Figure
FiO_2_: Fraction of inspired oxygen
PEEP: Positive end expiratory pressure
PR: Pulse rate
RA: Right atrium
RR: Respiratory rate
RV: Right ventricle
SpO_2_: Oxygen spirometry
Supp: Supplementary
TEE: Transesophageal echocardiography
TTE: Transthoracic echocardiography
TV: Tidal volume

## Declarations

### Declaration

We declare that this manuscript depicts the clinical management of patient with posterior clot post-CABG. Contributions from respective authors have been explicitly mentioned in the respective segment. This work has not been submitted to any other publication for publishing.

## References

[1] Nguyen HS, Nguyen HD, Vu TD (2018). Pericardial effusion following cardiac surgery. A single-center experience. Asian Cardiovasc Thorac Ann.

[2] Kuvin JT, Harati NA, Pandian NG, Bojar RM, Khabbaz KR (2002). Postoperative cardiac tamponade in the modern surgical era. Ann Thorac Surg.

[3] Ashikhmina EA, Schaff HV, Sinak LJ, Li Z, Dearani JA, Suri RM, Park SJ, Orszulak TA, Sundt TM (2010). Pericardial effusion after cardiac surgery: risk factors, patient profiles, and contemporary management. Ann Thorac Surg.

[4] Khan NK, Järvelä KM, Loisa EL, Sutinen JA, Laurikka JO, Khan JA (2017). Incidence, presentation and risk factors of late postoperative pericardial effusions requiring invasive treatment after cardiac surgery. Interact Cardiovasc Thorac Surg.

[5] Russo AM, O'Connor WH, Waxman HL (1993). Atypical presentations and echocardiographic findings in patients with cardiac tamponade occurring early and late after cardiac surgery. Chest.

[6] Chuttani K, Tischler MD, Pandian NG, Lee RT, Mohanty PK (1994). Diagnosis of cardiac tamponade after cardiac surgery: relative value of clinical, echocardiographic, and hemodynamic signs. Am Heart J.

[7] Price S, Prout J, Jaggar SI, Gibson DG, Pepper JR (2004). 'Tamponade' following cardiac surgery: terminology and echocardiography may both mislead. Eur J Cardiothorac Surg.

[8] Gabaldo K, Sutlić Ž, Miškovic D, Knežević Praveček M, Prvulović Đ, Vujeva B, Cvitkušic Lukenda K, Hadžibegović I (2019). Postpericardiotomy syndrome incidence, diagnostic and treatment strategies: experience AT two collaborative centers. Acta Clin Croat.

[9] Imazio M, Adler Y (2013). Management of pericardial effusion. Eur Heart J.

[10] Pepi M, Muratori M, Barbier P, Doria E, Arena V, Berti M, Celeste F, Guazzi M, Tamborini G (1994). Pericardial effusion after cardiac surgery: incidence, site, size, and haemodynamic consequences. Br Heart J.

[11] Ionescu A, Wilde P, Karsch KR (2001). Localized pericardial tamponade: difficult echocardiographic diagnosis of a rare complication after cardiac surgery. J Am Soc Echocardiogr.

[12] Imren Y, Tasoglu I, Oktar GL (2008). The importance of transesophageal echocardiography in diagnosis of pericardial tamponade after cardiac surgery. J Card Surg.

[13] Arntfield R (2019) Lifesaving applications of transesophageal echocardiography in critical and emergency care. ICU Management & Practice 3. https://www.sonosite.com/sites/default/files/Life%20Saving%20Applications%20of%20TEE%20in%20Critical%20and%20Emergency%20Care%2C%20November%202019.pdf. Accessed 9 Mar 2021.

[14] O’Neil M, Nagdev A, Teran F (2020) How to perform resuscitative transesophageal echocardiography in the emergency department. ACEP Now, Vol 39-No 07. https://www.intelligentultrasound.com/content/uploads/2020/09/How-to-Perform-Resuscitative-Transesophageal-Echocardiography-in-the-Emergency-Department-ACEP-Now.pdf. Accessed 9 Mar 2021.

[15] Heiberg J, El-Ansary D, Royse CF, Royse AG, Alsaddique AA, Canty DJ (2016). Transthoracic and transoesophageal echocardiography: a systematic review of feasibility and impact on diagnosis, management and outcome after cardiac surgery. Anaesthesia.

[16] Arntfield R, Pace J, McLeod S, Granton J, Hegazy A, Lingard L (2015). Focused transesophageal echocardiography for emergency physicians-description and results from simulation training of a structured four-view examination. Crit Ultrasound J.

[17] Fair J, Mallin M, Mallemat H, Zimmerman J, Arntfield R, Kessler R, Bailitz J, Blaivas M (2018). Transesophageal echocardiography: guidelines for point-of-care applications in cardiac arrest resuscitation. Ann Emerg Med.

[18] Teran F, Dean AJ, Centeno C, Panebianco NL, Zeidan AJ, Chan W, Abella BS (2019). Evaluation of out-of-hospital cardiac arrest using transesophageal echocardiography in the emergency department. Resuscitation.

[19] Arntfield R, Pace J, Hewak M, Thompson D (2016). Focused transesophageal echocardiography by emergency physicians is feasible and clinically influential: Observational results from a novel ultrasound program. J Emerg Med.

[20] Leichtle SW, Singleton A, Singh M, Griffee MJ, Tobin JM (2017). Transesophageal echocardiography in the evaluation of the trauma patient: A trauma resuscitation transesophageal echocardiography exam. J Crit Care.

[21] Osman A, Fong CP, Wahab SFA, Panebianco N, Teran F (2020). Transesophageal echocardiography at the golden hour: identification of blunt traumatic aortic injuries in the Emergency Department. J Emerg Med.

[22] Wray TC, Schmid K, Braude D, Azevedo K, Dettmer T, Tawil I, Boivin M, Marinaro J (2021). Safety of transesophageal echocardiography performed by intensivists and emergency physicians in critically ill patients with coagulopathy and thrombocytopenia: a single-center experience. J Intensive Care Med.

[23] Raiten J, Ahmed N, Amatya A, Sharma A, Acharya S, Lanahan J, Werlhof H, Ko HA, Tsui C, Reza T, Bajracharya S, Hagen O, Shrestha G (2020). Perioperative point-of-care ultrasound and transesophageal echocardiography in resource-limited settings-a focus on Nepal and Bangladesh. J Cardiothorac Vasc Anesth.

[24] Carmona P, Mateo E, Casanovas I, Peña JJ, Llagunes J, Aguar F, De Andrés J, Errando C (2012). Management of cardiac tamponade after cardiac surgery. J Cardiothorac Vasc Anesth.

[25] Reddy PS, Curtiss EI, O'Toole JD, Shaver JA (1978). Cardiac tamponade: hemodynamic observations in man. Circulation.

